# Efficacy and safety of JAK inhibitors in patients aged > 60 years with moderate-to-severe atopic dermatitis: a 52-week multicenter, real-life study—IL AD (Italian Landscape Atopic Dermatitis)

**DOI:** 10.1007/s00403-025-04278-9

**Published:** 2025-05-23

**Authors:** Luca Potestio, Cataldo Patruno, Alessandra Narcisi, Antonio Costanzo, Luciano Ibba, Luigi Gargiulo, Piergiorgio Malagoli, Michela Ortoncelli, Simone Ribero, Luca Mastorino, Francesco Leo, Silvia Mariel Ferrucci, Luisa Angileri, Francesca Barei, Luca Stingeni, Katharina Hansel, Claudio Sciarrone, Giampiero Girolomoni, Martina Maurelli, Caterina Foti, Benedetta Tirone, Anna Balato, Maria Esposito, Giovanni Paolino, Santo Raffaele Mercuri, Elena Pezzolo, Paola Savoia, Claudio Brescia, Maddalena Napolitano

**Affiliations:** 1 Section of Dermatology, Department of Clinical Medicine and Surgery, University of Naples Federico II, Via Pansini 5, 80131 Naples, Italy; 2 Department of Medicine and Health Sciences “Vincenzo Tiberio”, University of Molise, Campobasso, Italy; 3 Dermatology Unit, IRCCS Humanitas Research Hospital, Milan, Italy; 4 Department of Biomedical Sciences, Humanitas University, Milan, Italy; 5 Department of Dermatology, Dermatology Unit, Azienda Ospedaliera San Donato Milanese, Milan, Italy; 6 Departement of Clinical Medicine, Dermatologic Clinic, University of Turin, Turin, Italy; 7 Dermatology Unit. Fondazione, IRCCS Cà GRanda Ospedale Maggiore Policlinico Of Milan, Milan, Italy; 8 Dermatology Section, Department of Medicine and Surgery, University of Perugia, Perugia, Italy; 9 Department of Dermatology, Papardo Hospital, Messina, Italy; 10 Department of Medicine, Section of Dermatology and Venereology, University of Verona, Verona, Italy; 11 Section of Dermatology and Venereology, Department of Precision and Regenerative Medicine and Ionian Area (DiMePRe-J), University of Bari “Aldo Moro”, Bari, Italy; 12 Unit of Dermatology, University of Campania Luigi Vanvitelli, Naples, Italy; 13 Department of Biotechnological and Applied Clinical Sciences, University of L’Aquila, L’Aquila, Italy; 14 Unit of Dermatology, IRCCS Ospedale San Raffaele, Milan, Italy; 15 Dermatology Unit, Ospedale San Bortolo, Vicenza, Italy; 16 Department of Health Sciences, University of Eastern Piedmont, Novara, Italy

**Keywords:** Atopic dermatitis, Management, JAK inhibitors, Elderly patients, Abrocitinib, Baricitinib, Upadacitinib

## Abstract

Atopic dermatitis (AD) prevalence in elderly patients is increasing. Clinically, elderly AD may present with atypical phenotypes, making the diagnosis difficult. Moreover, treatment challenges arise due to treatment-resistance, comorbidities, polypharmacy, and contraindications to existing therapies. Janus kinase (JAK) inhibitors (abrocitinib, baricitinib, upadacitinib) may offer a valuable alternative. However, their use in elderly populations remains unclear, as older patients are often excluded from clinical trials, and several concerns have been raised about their safety in this category of subjects. This study aimed to evaluate the efficacy and safety of JAK inhibitors in elderly patients with moderate-to-severe AD. A 52-week, multicenter, real-life study was performed enrolling patients aged ≥ 60 years affected by moderate-to-severe AD undergoing treatment with JAK inhibitors for at least 16 weeks across 16 dermatological centers in Italy. Disease severity was assessed at baseline, week (W) 4, 16, 24, and 52 using the Eczema Area and Severity Index (EASI), Dermatology Life Quality Index (DLQI), and Pruritus-Numerical Rating Scale (P-NRS). Adverse events (AEs) were recorded at each follow-up. A total of 72 patients met the inclusion criteria [abrocitinib: 13 (18.06%); baricitinib: 6 (8.33%); upadacitinib: 53 (73.61%)]. Of these, 72 (100.0%) achieved W16 follow-up with 33 (45.83%) and 26 (36.11%) subjects reaching W24 and W52, respectively. At baseline, mean EASI, DLQI and P-NRS were 21.22 ± 10.38, 18.21 ± 7.33, and 7.84 ± 1.94, respectively. A significant improvement in all scores was observed starting from W4 [EASI: 4.77 ± 5.07, DLQI: 4.01 ± 3.98, P-NRS: 1.66 ± 1.83 (p < 0.0001 for all)], continuing to improve up to W52 [EASI: 0.81 ± 1.27, DLQI: 0.31 ± 0.63, P-NRS: 0.42 ± 1.03; (p < 0.0001 for all)]. No treatment interruptions or modifications for ineffectiveness or AEs were registered. No statistically significant differences in terms of efficacy and safety were found among the treatment groups. JAK inhibitors demonstrated significant efficacy and an acceptable safety profile in elderly AD patients.

## Introduction

Atopic dermatitis (AD) is a chronic, inflammatory skin disease, characterized by eczema and itch, typically affecting the face, neck, and flexures of the limbs depending on the age of the patient [1, 2]. While AD was traditionally regarded as a common childhood condition, recent epidemiological research has shown that it is also prevalent among adults and the elderly [3]. Although epidemiological data on AD prevalence in older adults are limited, it has been estimated to range from 7.0 to 9.3% among subjects aged 75–99 [4]. Clinically, elderly AD is recognized as a distinct subtype, as it may present with atypical clinical phenotypes, including generalized eczema, prurigo nodularis, and nummular eczema, occurring more frequently than in other age groups [6, 7]. Consequently, clinical diagnosis may be difficult [6, 7]. It should also be underlined that moderate-to-severe AD seems to be more frequent in older adults as compared with younger subjects [4].

The treatment can be challenging, especially in moderate-to-severe cases in which therapy with conventional systemic immunosuppressive drugs (e.g. cyclosporine, methotrexate, azathioprine, mycophenolate) may be challenging due to frequent comorbidities in older patients, potential drug interactions, and reduced physiological resilience [8, 9]. Currently available biologics for AD (dupilumab, tralokinumab, lebrikizumab), are characterized by a high profile in terms of safety [10–13]. Literature data on their use in the elderly are increasing [10–13]. However, there are patients who are unresponsive to these drugs, and in rare cases, there may be no suitable alternatives due to contraindications such as hypersensitivity or needle-phobia. It should be pointed out that no drug is 100% effective in all patients, regardless of age, and that any drug can cause adverse events of varying severity [14].

In this context, Janus kinase (JAK) inhibitors may represent a valuable option. Currently, three JAK inhibitors have been approved for AD management: abrocitinib (JAK1 selective inhibitor), baricitinib (JAK1 and JAK2 inhibitor), and upadacitinib (selective and reversible JAK1 inhibitor) [9].

However, the European Medicines Agency (EMA) raised concerns about their use in older patients (aged ≥ 65 years), recommending their usage only if no suitable treatment alternatives are available [15]. Moreover, oral JAK inhibitors have a black box warning on increased risk for developing serious, life-threatening infections, malignancies, and cardiovascular events [16, 17]. It should be also considered that elderly patients are often excluded from clinical trials [18]. In detail, patients aged more than 75 years were excluded from registration trial for abrocitinib (JADE MONO-1, JADE MONO-2), baricitinib (BREEZE-AD) and upadacitinib (MEASURE UP 1, MEASURE UP 2, AD UP) [18]. Real-life data are mandatory to better evaluate the effectiveness and safety of these drugs in this population, although limited by the need to follow national regulations. To date, data for the use of JAK inhibitors in elderly patients affected by AD are limited to case series and case reports [19, 20].

The aim of our study was to evaluate the effectiveness and safety of JAK inhibitors (abrocitinib, baricitinib and upadacitinib) for the management of AD patients in elderly subjects. For the purpose of this study, we defined ‘elderly’ as patients aged ≥ 60 years.

## Material and methods

A 52-week, multicenter, retrospective, real-life study was performed enrolling patients aged ≥ 60 years affected by moderate-to-severe AD undergoing treatment with JAK inhibitors. Data were collected from 16 dermatological referral centers evenly distributed in Northern, Central, and Southern Italy. Inclusion criteria were as follows: age ≥ 60 years; diagnosis of AD made by a dermatologist; treatment with a JAK inhibitor and had completed at least 16 weeks of follow-up at the time of data analysis; criteria for treatment with JAK inhibitors according to prescription guidelines issued by the Italian Medical Agency. Of note, a threshold of ≥ 60 years to define ‘elderly’ patients has been chosen, in line with previous dermatologic studies and real-life clinical experience, despite the EMA using ≥ 65 as a cut-off for specific safety concerns. Our goal was to provide data on a broader aging population, including those often underrepresented in clinical trials.

All patients were allowed to use standard-of-care topical therapies (e.g., emollients, topical corticosteroids, or calcineurin inhibitors) as needed during JAK inhibitor treatment.

Patients with comorbidities representing contraindications to treatment with JAK inhibitors (active malignancy or history of malignancy within the last five years, history of thromboembolic events, active or latent tuberculosis, and any other condition deemed a contraindication to JAK inhibitor therapy according to EMA guidance), as well as patients receiving drugs with potential interactions with JAK inhibitors were excluded. An additional inclusion criterion was that all other available treatment options for AD (topicals, systemic immunosuppressants, biologics) had been either ineffective, contraindicated, or not tolerated. In detail, biologics were attempted prior to JAK inhibitors in eligible patients. However, in some cases (e.g., needle-phobia, hypersensitivity, or patient preference), initiation of biologic therapy was not feasible. Thus, patients who had not received biologics met the inclusion criterion of having failed or being ineligible for all other approved treatments. At baseline, the following clinical and demographic data were collected: age, sex, body mass index, medical history, clinical phenotype of AD, atopic comorbidities, and previous systemic treatment history for AD. AD severity was evaluated at baseline and at each follow-up [week (W) 4, 16, 24, and 52] by using Eczema Area and Severity Index (EASI), Dermatology Life Quality Index (DLQI), and Pruritus-Numerical Rating Scale (P-NRS). Any new clinical symptoms reported by the patient or observed during physical examinations were recorded at each visit to identify possible adverse events (AEs). The causal relationship between reported adverse events and JAK inhibitors was assessed by investigators at each center, based on temporal correlation, plausibility, and exclusion of alternative causes, following standard pharmacovigilance principles. Clinical improvement and safety data were also analyzed for each specific drug (abrocitinib, baricitinib and upadacitinib) to highlight any differences.

It is important to note that the absence of data at W24 and 52 for some patients was not due to treatment discontinuation or dropout. Instead, these patients continued their JAK inhibitor therapy but were not yet evaluable at later timepoints due to the observational nature of the study and varying follow-up durations across participating centers.

Categorical variables were reported as frequencies and percentages, whereas continuous variables were expressed as mean ± standard deviation (SD). The statistical significance of clinical improvement in EASI, P-NRS, and DLQI scores at W4, W16, W24, and W52 compared to baseline was assessed using Student’s t-test. All statistical analyses were conducted using GraphPad Prism software (v.8.0; GraphPad Software Inc., La Jolla, CA, USA), with a significance threshold set at p < 0.05.

## Results

A total of 87 patients were enrolled in the study. Of the 87 patients initially considered, 15 (17.24%) were excluded due to comorbidities contraindicating JAK inhibitor therapy (n = 5), concomitant medications with potential for significant drug-drug interactions (n = 3), insufficient treatment duration (< 16 weeks at time of analysis; n = 7). Seventy-two (82.76%) [36 males (50.0%); mean age 68.83 ± 6.95 years (range 60–87)] met the inclusion criteria. Clinical and demographic data have been summarized in Table 1. In detail, 26 (36.11%) patients were aged 60–64 years, 17 (23.61%) were aged 65–69 years, 13 (18.06%) were aged 70–74 years, 10 (13.89%) were aged 75–79 years, and 6 (8.33%) were aged > 80 years. Thirteen (18.06%), 6 (8.33%), and 53 (73.61%) subjects were treated with abrocitinib [50 mg: 1 (7.69%); 100 mg: 12 (92.31%)], baricitinib [2 mg: 0 (0%); 4 mg: 6 (100.0%)], or upadacitinib [15 mg: 37 (69.81%); 30 mg: 16 (30.19%)], respectively. Notably, upadacitinib was administered at 15 mg (n = 37) or 30 mg (n = 16). In patients aged ≥ 65, the 30 mg dosage was prescribed in selected cases based on disease severity and specialist judgment, despite 15 mg being the EMA-approved dose in this age group. Clinical features for each drug were summarized in Table 2.

**Table 1:** Demographic data and clinical outcomes of the study population

Study Population	
Number of patients	72
Sex, male	36 (50.0%)
Mean age	68.83 ± 6.95
Body mass index	25.9 ± 3.76
Age of AD onset (years)	51.09 ± 21.25
Mean duration of AD (years)	17.72 ± 19.70
History of atopy	12 (16.67%)
AD phenotype Lichenified/exudative flexural dermatitis Nummular dermatitis Generalized eczema Prurigo nodularis like Erythroderma Hand eczema	45 (62.50%) 4 (5.56%) 10 (13.89%) 10 (13.89%) 1 (1.39%) 2 (2.78%)
Atopic comorbidities Rhinitis Asthma Conjunctivitis	10 (13.89%) 7 (9.72%) 5 (6.94%)
Previous conventional treatments Oral corticosteroids Cyclosporine Methotrexate Phototherapy Dupilumab Tralokinumab Abrocitinib Baricitinib Upadacitinib	25 (34.72%) 20 (27.78%) 5 (6.94%) 2 (2.78%) 30 (41.67%) 6 (8.33%) 0 0 2 (2.78%)
Current treatment Abrocitinib Baricitinib Upadacitinib	13 (18.06%) 6 (8.33%) 53 (73.61%)
Baseline (number of patients) EASI DLQI P-NRS	72 21.22 ± 10.38 18.21 ± 7.33 7.84 ± 1.94
Week 4 (number of patients) EASI DLQI P-NRS	72 (100.0%) 4.77 ± 5.07* 4.01 ± 3.98* 1.66 ± 1.83*
Week 16 (number of patients) EASI DLQI P-NRS	72 (100.0%) 1.75 ± 3.49* 1.14 ± 2.17* 0.55 ± 1.19*
Week 24 (number of patients) EASI DLQI P-NRS	33 (45.83%) 1.24 ± 2.31* 0.75 ± 1.06* 0.50 ± 0.98*
Week 52 (number of patients) EASI DLQI P-NRS	26 (36.11%) 0.81 ± 1.27* 0.31 ± 0.63* 0.42 ± 1.03*

*AD* atopic dermatitis, *EASI* eczema area severity index, *DLQI* dermatology life quality index, *P-NRS* pruritus-numerical rating scale

^*^: p < 0.0001 if compared to the same score at baseline

**Table 2:** Demographic data and clinical outcomes of patients receiving abrocitinib, baricitinib, and upadacitinib,

	**Abrocitinib**	**Baricitinib**	**Upadacitinib**
Number of patients	13	6	53
Sex, male	8 (61.54%)	2 (33.33%)	26 (49.06%)
Mean age	68.62 ± 5.78	69.67 ± 9.31	68.79 ± 7.06
Body mass index	24.75 ± 2.45	25.48 ± 3.14	26.16 ± 4.02
Age of AD onset (years)	51.69 ± 17.24	45.33 ± 34.7	51.63 ± 20.64
Mean duration of AD (years)	16.92 ± 16.30	24.33 ± 34.05	17.12 ± 18.68
History of atopy	3 (23.08%)	1 (16.67%)	8 (15.09%)
AD phenotype Lichenified/exudative flexural dermatitis Nummular dermatitis Generalized eczema Prurigo nodularis like Erythroderma Hand eczema	11 (84.62%) 0 1 (7.69%) 1 (7.69%) 0 0	5 (83.33%) 1 (16.67%) 0 0 0 0	29 (54.72%) 4 (7.55%) 7 (13.21%) 8 (15.09%) 1 (1.89%) 2 (3.77%)
Atopic comorbidities Rhinitis Asthma Conjunctivitis	2 (15.38%) 1 (7.69%) 0	1 (16.67%) 0 1 (16.67%)	7 (13.21%) 6 (11.32%) 4 (7.55%)
Previous conventional treatments Oral corticosteroids Cyclosporine Methotrexate Phototherapy Dupilumab Tralokinumab Abrocitinib Upadacitinib Baricitinib	6 (46.15%) 5 (38.46%) 1 (7.69%) 1 (7.69%) 7 (53.85%) 4 (30.77%) 0 2 (15.38%) 0	0 0 0 0 2 (33.33%) 0 0 0 0	19 (35.85%) 15 (28.30%) 4 (7.55%) 1 (1.89%) 21 (39.62%) 2 (3.77%) 0 0 0
Drug dosage	50 mg: 1 (7.69%) 100 mg: 12 (92.31%)	2 mg: 0 4 mg: 6	15 mg: 37 (69.81%) 30 mg: 16 (30.19%)
Baseline (number of patients) EASI DLQI P-NRS	13 20.46 ± 9.42 18.50 ± 4.43 7.31 ± 1.49	6 23.60 ± 10.47 22.17 ± 5.08 9.17 ± 0.75	53 21.13 ± 10.74 17.30 ± 7.24 7.85 ± 2.06
Week 4 (number of patients) EASI DLQI P-NRS	13 (100.0%) 5.47 ± 4.36* 4.24 ± 3.56* 2.27 ± 1.39*	6 (100.0%) 4.33 ± 1.37° 4.17 ± 3.18* 2.17 ± 1.47*	53 (100.0%) 4.45 ± 5.13° 3.05 ± 3.39* 1.46 ± 1.83*
Week 16 (number of patients) EASI DLQI P-NRS	13 (100.0%) 2.88 ± 3.44* 1.67 ± 2.16* 0.88 ± 0.99*	6 (100.0%) 1.33 ± 1.21°* 0.83 ± 1.39* 0.67 ± 0.81*	53 (100.0%) 1.54 ± 3.53* 0.56 ± 0.98* 0.49 ± 1.25*
Week 24 (number of patients) EASI DLQI P-NRS	6 (46.15%) 1.33 ± 2.42* 1.33 ± 1.36* 0.71 ± 1.25*	No data	27 (50.94%) 1.22 ± 2.33* 0.46 ± 0.66* 0.38 ± 0.75*
Week 52 (number of patients) EASI DLQI P-NRS	3 (23.08%) 0.67 ± 0.58* 0 ± 0* 0.67 ± 0.58*	No data	23 (43.40%) 0.78 ± 1.24* 0.33 ± 0.65* 0.48 ± 1.08*

*AD* atopic dermatitis, *EASI* eczema area severity index, *DLQI* dermatology life quality index, *P-NRS* pruritus-numerical rating scale

°: p < 0.01 if compared to the same score at baseline

°*: p < 0.001°* if compared to the same score at baseline

^*^: p < 0.0001 if compared to the same score at baseline

Mean age of AD onset was 51.09 ± 21.25 years, with a mean duration of the disease of 17.72 ± 19.70 years. Regarding clinical presentation, lichenified/exudative flexural dermatitis was the common form (n = 45, 62.50%), followed by generalized eczema (n = 10, 13.89%), and prurigo nodularis (n = 10, 13.89%). The most frequently reported atopic comorbidity was rhinitis (n = 10, 13.89%), followed by asthma (n = 7, 9.72%), and conjunctivitis (n = 5, 6.94%).

Dupilumab was the most common previous systemic drug used for AD management (n = 30, 41.67%) [main reasons for discontinuation: inadequate clinical response (n = 22), AEs such as conjunctivitis or injection site reactions (n = 4), and patient preference or logistical factors (n = 4)], followed by oral corticosteroids (n = 25, 34.72%) and cyclosporine (n = 20, 27.78%).

Most patients did not present with multiple or severe comorbidities. The most commonly reported comorbid conditions were arterial hypertension (n = 10, 13.89%), diabetes mellitus (n = 2, 2.78%), dyslipidaemia (n = 2, 2.78%), psoriatic arthritis (n = 2, 2.78%), psoriasis (n = 1, 1.39%), rheumatoid arthritis (n = 1, 1.39%), anxious disorder (n = 1, 1.39%), coeliac disease (n = 1, 1.39%), chronic gastritis (n = 1, 1.39%), hyperuricemia (n = 1, 1.39%), and osteoporosis (n = 1, 1.39%). Follow-up data at W16 were available for all included patients (n = 72, 100.0%), as this was a pre-specified inclusion criterion, whereas W24 and W52 follow-up were only available for 33 [45.83% (abrocitinib: 6; baricitinib: 0; upadacitinib: 27)] and 26 [(36.11%), (abrocitinib: 3; baricitinib: 0; upadacitinib: 23)] subjects, respectively.

**Fig. 1:**
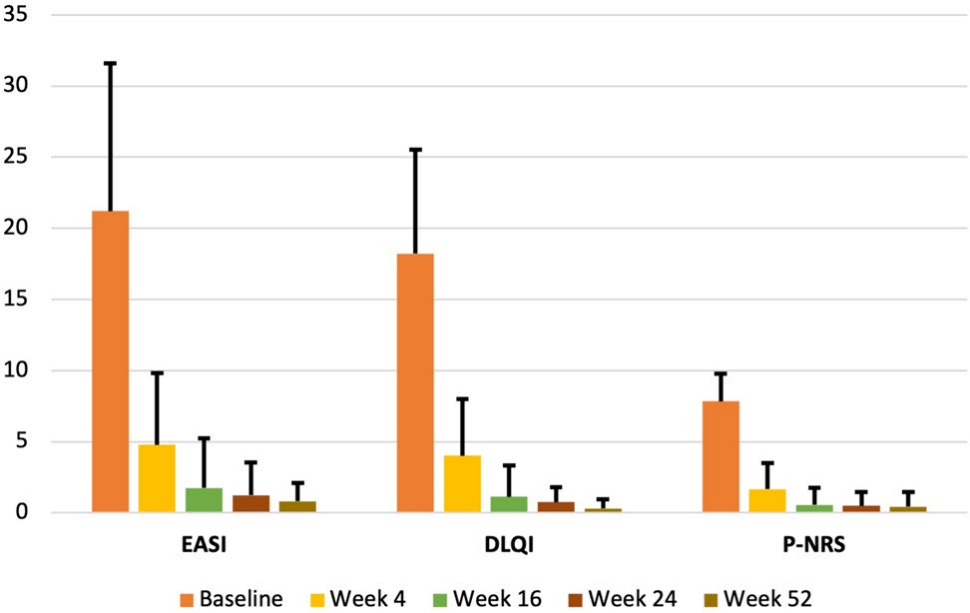
Clinical outcomes over time (EASI, DLQI, P-NRS) of the study population. *EASI* eczema area severity index, *DLQI* dermatology life quality index, *P-NRS* pruritus-numerical rating scale. Y-axis: mean ± standard deviation for EASI, DLQI, and P-NRS

The mean EASI score at baseline was 21.22 ± 10.38 and was significantly and progressively reduced at W4 (4.77 ± 5.07, p < 0.0001), W16 (1.75 ± 3.49, p < 0.0001). W24 (1.24 ± 2.31, p < 0.0001), and W52 (0.81 ± 1.27, p < 0.0001) (Fig. 1).

DLQI also significantly reduced from a baseline of 18.21 ± 7.33, to 4.01 ± 3.98 at W4, 1.14 ± 2.17 at W16, 0.75 ± 1.06 at W24, and 0.31 ± 0.63 at W52 (p < 0.0001 for all the time points) (Fig. 1).

Finally, P-NRS at baseline was 7.84 ± 1.94, improving to 1.66 ± 1.83, 0.55 ± 1.19, 0.50 ± 0.98, and 0.42 ± 1.03 at W4, W16, W24 and W52, respectively (p < 0.0001 for all) (Fig. 1).

At W4, 66.67% (48/72) and 36.11% (26/72) of patients achieved EASI 75 and EASI 90, respectively. At W16, these proportions increased to 87.50% (63/72) and 70.83% (51/72). At W24, 90.91% (30/33) achieved EASI 75 and 81.82% (27/33) achieved EASI 90. At W52, EASI 75 and EASI 90 were achieved by 92.31% (24/26) and 88.46% (23/26), respectively.

**Fig. 2:**
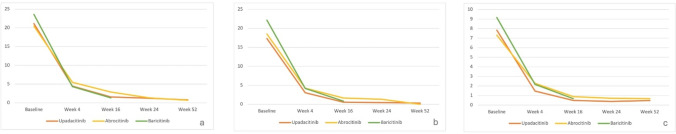
Drug specific outcomes (abrocitinib, baricitinib, upadacitinib) for EASI (**a**), DLQI (**b**), and P-NRS (**c**). *EASI* eczema area severity index, *DLQI* dermatology life quality index, *P-NRS* pruritus-numerical rating scale. Y-axis: mean value for EASI (**a**), DLQI (**b**), and P-NRS (**c**)

No cases of treatment interruption due to ineffectiveness were reported. Specifically, no statistically significant differences in terms of effectiveness were found among the treatment groups, with a significant improvement reported for all the investigated scores from baseline to each timepoint (Table 2, Fig. 2).

Sub-analysis of treatment response among the various AD phenotypes or age subgroups found no significant differences. Moreover, a subgroup analysis comparing patients previously treated with dupilumab (n = 30) versus those who were naïve to biologics showed no statistically significant differences in clinical response at any time point.

As regards safety, the following AEs were collected: hypercholesterolemia (n = 4, 5.56%), nausea (n = 4, 5.56%), arthralgia (n = 1, 1.39%), headache (n = 1, 1.39%), erectile dysfunction (n = 1, 1.39%), myalgia (n = 1, 1.39%), genital herpes reactivation (n = 1, 1.39%), and leukopenia (n = 1, 1.39%).

Specifically, the majority of AEs were mostly observed in the upadacitinib cohort, with just 1 case of hypercholesterolemia in the abrocitinib group. However, no cases of treatment interruption for AEs were reported, as well as no dosage reduction in patients receiving abrocitinib 100 mg (n = 12, 92.31%), baricitinib 4 mg (n = 6, 100%), or upadacitinib 30 mg (n = 16, 30.19%) were observed.

## Discussion

Despite the growing elderly population affected by AD, data on JAK inhibitors remain limited due to trial exclusions and safety concerns. Real-life studies are essential to understand their use in this setting. Currently, only one case series involving 7 elderly patients affected by AD successfully treated with upadacitinib [18], and a case series on 3 elderly patients affected by eczema (n = 2) and lichen amyloidosis (n = 1) successfully treated with baricitinib have been reported [20].

Our study was the first to evaluate the effectiveness and safety of currently available JAK inhibitors (abrocitinib, baricitinib and upadacitinib) for the management of AD in patients aged ≥ 60 years. A total of 72 patients were enrolled. All the subjects (n = 72 100.0%) achieved W16 of follow-up, whereas W24 and W52 follow-up were only available for 33 (45.83%) and 26 (36.11%) subjects, respectively. A significant reduction in all the investigated scores was observed starting at W4 and confirmed at each follow-up up to W52.

Specifically, 13 (18.06%), 6 (8.33%), and 53 (73.61%) patients were treated with abrocitinib, baricitinib and upadacitinib, respectively. A significant improvement for EASI, DLQI and P-NRS from baseline to each timepoint was reported for each treatment group. No cases of treatment interruption for ineffectiveness were reported. Finally, no statistically significant differences in terms of effectiveness were found among the treatment groups. However, due to the highly unequal sample sizes, particularly the small baricitinib cohort (n = 6), statistical comparisons among treatment groups were considered exploratory and should be interpreted with caution.

Regarding the safety, no cases of treatment interruption or dosage reduction for AEs were reported. Of note, most AEs were observed in the upadacitinib group, which represented the majority of treated patients in our cohort. Therefore, the higher absolute number of AEs is likely attributable to the larger sample size. Nevertheless, we cannot exclude the potential influence of age-related pharmacokinetic changes in elderly patients, which may affect drug metabolism and tolerability. Furthermore, although clinical response to JAK inhibitors was evident, the limited AE reporting in our cohort, particularly the absence of common AEs such as upper respiratory infections, likely reflects the retrospective design. Prospective studies are needed to capture a more comprehensive safety profile in elderly patients.

Finally, it should be highlighted that the absence of treatment discontinuation is unusual as compared to previous studies [23, 24] and must be interpreted with caution. It likely reflects a combination of patient selection, exclusion of early dropouts (< 16 weeks), and potential site-related reporting factors. Larger studies with longer follow-up are needed to verify this finding.

Main strengths of our study include data accuracy, the comparability of baseline features of the study cohorts, and the focus of elderly patients. Main limitations of the study are the retrospective design, the reduced number of patients achieving W24 and W52 follow-up (related to differences in data availability across centers rather than treatment discontinuation), the exclusion of patients with significant comorbidities, and the different sample size between abrocitinib, baricitinib and upadacitinib cohorts, which limit the ability to draw firm conclusions regarding comparative efficacy and safety. Furthermore, the exclusion of patients with < 16 weeks of follow-up may have led to an overestimation of treatment retention and efficacy, as early discontinuations due to inefficacy or AEs could not be captured. Although our rationale was to ensure sufficient exposure for assessment, we recognize that this choice introduces a positive selection bias. Notably, the absence of baricitinib data at W24 and W52 was due to limited follow-up duration at the sites prescribing this agent, rather than treatment discontinuation. Nevertheless, this restricts direct comparisons of long-term efficacy across drugs and represents a limitation of the study. Finally, given the retrospective nature of the study, detailed data on topical medication use was not always available, which may have influenced the interpretation of clinical response. On consequence, these comparisons should be considered exploratory.

Our findings are particularly relevant for clinical practice, as they suggest that, when carefully selected and monitored, elderly patients may benefit from JAK inhibitors without increased safety concerns. This could provide a valuable therapeutic option in cases where biologics are ineffective or contraindicated, potentially expanding treatment access for a population often excluded from clinical trials. Interestingly, common AEs reported in younger populations treated with JAK inhibitors, such as acne, were not observed in our cohort. This may be due to the retrospective nature of the study and potential underreporting in clinical records, especially for mild or self-limited events. Alternatively, age-related differences in immune response or skin physiology might influence the incidence of certain AEs in elderly patients. Further studies are warranted to explore these differences.

Our results confirmed the effectiveness of these drugs in this population and also supported their favourable safety profile, provided that patients who are suitable for JAK inhibitors and the dosage of drugs are carefully selected and sufficient attention is paid to the occurrence of side effects. Certainly, further studies are needed to confirm our data.

## Data Availability

No datasets were generated or analysed during the current study.
